# Low-Power Sensor Interface with a Switched Inductor Frequency Selective Envelope Detector

**DOI:** 10.3390/s21062124

**Published:** 2021-03-18

**Authors:** Marko Gazivoda, Vedran Bilas

**Affiliations:** Faculty of Electrical Engineering and Computing, University of Zagreb, 10000 Zagreb, Croatia; vedran.bilas@fer.hr

**Keywords:** sensor signal conditioning circuit, event detection application, switched inductor filter, weak signal detection

## Abstract

With the growing need to understand our surroundings and improved means of sensor manufacturing, the concept of Internet of Things (IoT) is becoming more interesting. To enable continuous monitoring and event detection by IoT, the development of low power sensors and interfaces is required. In this work we present a novel, switched inductor based acoustic sensor interface featuring a bandpass filter and envelope detector, perform a sensitivity, frequency selectivity, and power consumption analysis of the circuit, and present its design parameters and their qualitative influence on circuit characteristics. We develop a prototype and present experimental characterization of the interface and its operation with input signals up to 20 mV peak-to-peak, at low acoustic frequencies from 100 Hz to 1 kHz. The prototype achieves a sensitivity of approximately 2 mV/mV in the passband, a four times lower sensitivity in the stopband, and a power consumption of approximately 3.31 µW. We compare the prototype interface to an interface consisting of an active bandpass filter and a passive voltage doubler using a prerecorded speedboat signal.

## 1. Introduction

The growing need to understand and manage our surroundings, coupled with advances in sensor technologies and manufacturing processes [1], has led to an increased interest in the concept of Internet of Things (IoT), which envisions sensor networks consisting of hundreds of thousands of small, robust sensor nodes utilized to continuously monitor real-world events and processes [2,3,4]. Continuous monitoring and event detection emphasize the need for low-power sensors and sensor signal conditioning circuits which enable the node to achieve long life-times, even when powered by small batteries [3,4,5].

Acoustic sensors present an attractive choice for IoT applications because they generate signals that are rich in information and can be processed using relatively simple hardware [6,7,8] that powers up the rest of the sensor node only upon detection of an event of interest [4,5], thereby reducing the power consumption of an acoustic sensor node. These wake-up sensor interfaces utilize bandpass filtering, envelope detection, quantization, and some rudimentary form of classification to determine if an event of interest occurred. Implementations of the wake-up interface with an active bandpass filter, diode envelope detector, and microcontroller-based classification are presented in [9,10]. The power consumption of the bandpass filter and the envelope detector is reported as 8.25 µW in [9] and 20.74 µW in [10].

The envelope detector is one of the critical elements in the weak signal front ends in various applications (sensing, communications, energy harvesting) due to its power-consumption to sensitivity trade-off [11,12,13,14,15,16]. In [17] we studied the impact of the envelope detector on sensitivity and power consumption of the wake-up sensor interface in the lower audio frequency range. Based on the mechanically switched inductor energy harvester [16], in [18] we demonstrated that a piezoelectric energy harvester can be used as a vibration sensor utilizing a mechanically switched inductor driven by the sensed vibrations.

In order to increase the sensitivity of low-power acoustic wake-up sensor interfaces, and at the same time lower their power consumption, in this work we propose a novel approach, utilizing an electrically switched inductor as a replacement for conventionally used bandpass filter and envelope detector functional blocks. Using this approach, inspired by the switched inductor bandpass filter [19,20], and the switched inductor energy harvester [12,13,16], we devise a novel, low-power wake-up sensor interface, operational with weak input signals (around 5 mV) in the low acoustic frequency range (100 Hz–1 kHz) and applicable in low-power always-on acoustic event detectors.

With this work we present several contributions: a novel, frequency-selective, voltage-boosting, low-power, weak-signal acoustic sensor interface; a sensitivity, frequency selectivity and power consumption analysis of the circuit; design parameter selection, and their influence on interface characteristics; experimental characterization of a prototype, and its comparison to an interface consisting of an active bandpass filter and a passive voltage doubler.

The rest of this paper is organized as follows: Section 2 presents related circuits and principles of operation. Section 3 shows the proposed interface characteristics and design parameters. Section 4 presents a simulation study of the sensor interface, determining its key design parameters and desired functionality. Section 5 shows the developed prototype and its experimental characterization. In Section 6 a set of design recommendations for interface synthesis are given. Section 7 presents a comparison of the novel sensor interface and interface presented in [9,18] and Section 8 states the concluding remarks of the paper and presents future work.

## 2. Related Circuits and Principles of Operation

The proposed sensor interface utilizes the switched inductor for filtering the sensor signal and extracting and boosting its envelope. This concept was inspired by two previous lines of work, the switched inductor filter and the switched inductor energy harvester.

### 2.1. Switched Inductor Filter

**Figure 1:**
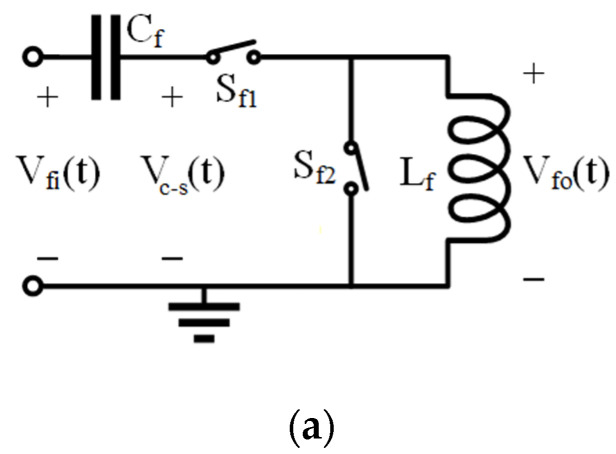

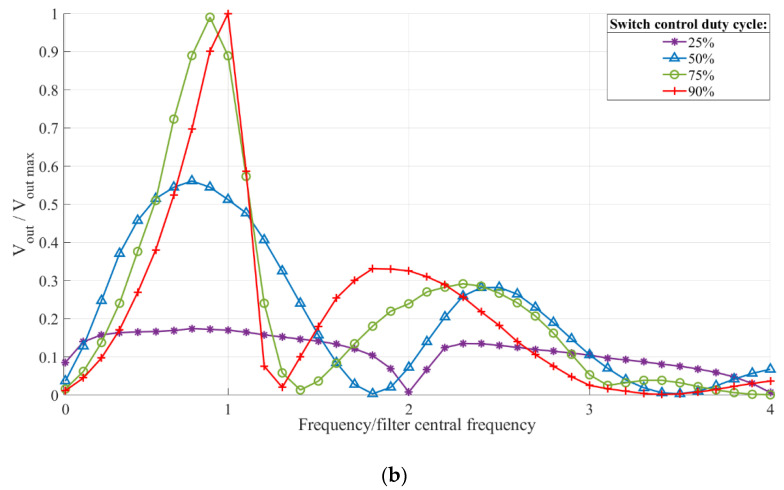
(**a**) Switched inductor filter and (**b**) its qualitative frequency characteristic with the duty cycle of 25% (purple), 50% (blue), 75% (green), and 90% (red). Filter output voltage RMS is normalized with regards to maximal output voltage RMS (obtained with the 90% duty cycle at the input frequency equal to filter central frequency), input signal frequency normalized with regards to the filter’s central frequency.

The switched inductor filter (shown in Figure 1a) consisting of a capacitor, *C_f_*, inductor, *L_f_*, and two switches *S_f_*_1_ and *S_f_*_2_, is used in power electronics to electrically tune the frequency characteristic of inverter outputs, suppressing unwanted harmonics [19,20]. Figure 1b shows the electrically tunable frequency characteristic of such a filter and the impact of the switch control function duty cycle as its tuning parameter. The input signal frequency was normalized with regards to the filter central frequency and the output voltage root mean square (RMS) was normalized with regards to the maximal filter output RMS voltage (obtained with the 90% duty cycle when the input signal frequency was equal to the filter central frequency).

The passive LC filter has a resonant frequency, *f_res_:*

(1) $$f_{res}=\frac{1}{2\pi \sqrt{L_{f}C_{f}}}$$

where *L_f_* and *C_f_* are the values of inductance and capacitance, respectively. By switching the inductor on and off, its effective value, *L_feff_*, seen at the circuit input, is changed, which changes the filter’s frequency characteristics. The two switches, *S_f_*_1_ and *S_f_*_2_, (Figure 1a) are driven by two antiparallel square signals, with switch *S_f_*_2_ closing when *S_f_*_1_ opens to provide a discharge current path for the inductor. The switching function *F*_1_*(t)* of the switch *S*_1_ is given as [20]:

(2) $$F_{1}\left(t\right)=A_{0}+2\overset{\infty }{\underset{n=1}{\sum }}\frac{\sin\left(n\omega \frac{d}{2}\right)}{n\pi }\cos\left(n\omega t-n\theta \right)$$

where *t* denotes time, *d* and *θ* are the pulse duration and the phase delay of the switch control function, respectively, ω is the angular frequency of the switch control signal, *n* is a positive integer, and *A_0_* is the average value of the switching function on its single period *T*. The average value of the switching function is determined by its duty cycle, i.e., the ratio of the duration of the function’s high state and its period, *A_0_* = *d*/*T*. The switching function takes on the value of 1 when the switch is closed and 0 when it is open.

The authors of [19,20] do not analyze the influence of the switch control function frequency on the filter functionality. They only state that it should be higher than input signal frequency *f_in_*.

The filter output voltage, *V_fo_(t)*, is determined by the voltage of the node between the filter capacitor *C_f_* and switch *S_f_*_1_, *V_c-s_(t)*, and the switching function *F*_1_*(t)*:

(3) $$V_{fo}\left(t\right)=F_{1}\left(t\right)\cdot V_{c-s}\left(t\right)$$

From Equations (2) and (3) and a few steps presented in [20], it can be determined that the effective value of the filter inductance *L_feff_* is proportional to:

(4) $$L_{feff}\propto \frac{L_{f}}{A_{0}{}^{2}}$$

and therefore, dependent on the average value of the switching function, which is, as shown previously, determined by the switching function’s duty cycle that can be used to tune the filter’s frequency characteristic, as shown in Figure 1b.

### 2.2. Switched Harvester on Inductor

**Figure 2:**
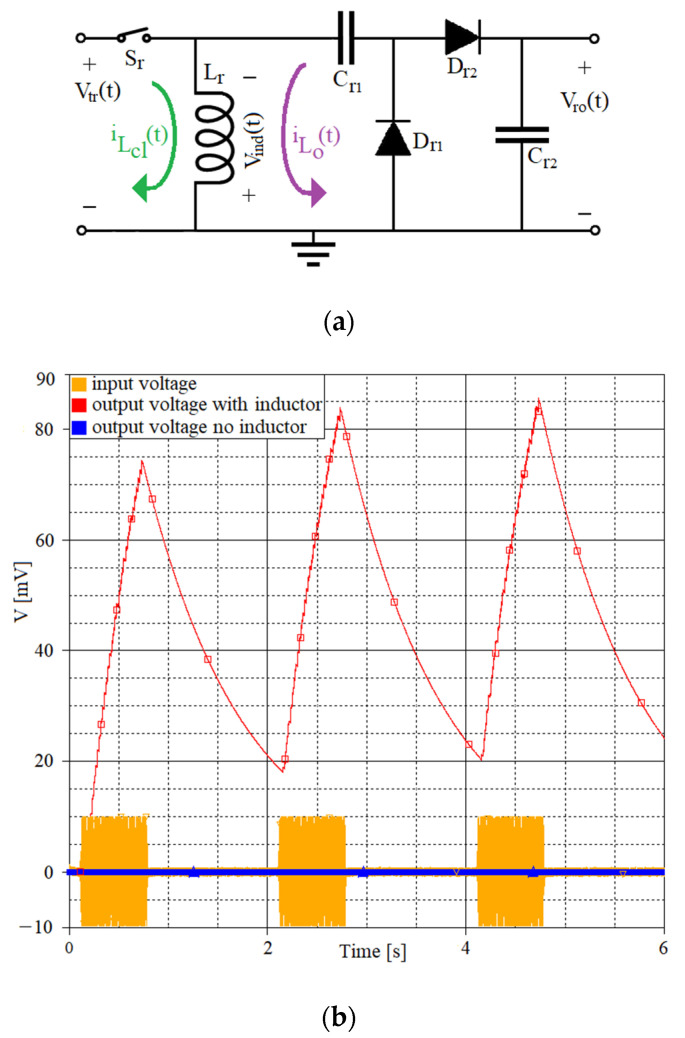
(**a**) Switched harvester on inductor with marked inductor current, *i_Lcl_(t)* (green) and *i_Lo_(t)* (purple) with the switch closed and opened, respectively. (**b**) Switched harvester on inductor output signal waveform (red) compared to a rectifier without the switched inductor (blue). Input signal (yellow): 500 ms of sinusoidal signal, 20 mV peak-to-peak, 100 Hz, followed by a 1.5 s pause. *C_r_*_1_ = *C_r_*_2_ = 1 µF, *L_r_* = 100 mH, switch control frequency *f_switch_* = 256 Hz, duty cycle 50%.

The switched harvester on inductor (one version shown in Figure 2a) is used to increase the efficiency in energy harvesting, by boosting the harvester’s transducer voltage, *V_tr_(t)*, prior to rectification (as shown in Figure 2b) [12,13,16].

While the switch *S_r_* is closed, the energy of the harvester’s transducer signal is stored in the magnetic field of the inductor, *L_r_*, changing the inductor’s current by Δ*i_L_* with:

(5) $$\Delta i_{L}=\frac{1}{L_{r}}\int_{t_{1}}^{t_{2}}V_{tr}\left(t\right)dt$$

where *t*_1_ and *t*_2_ are, respectively, the beginning and ending moment of observing the storing of energy in the inductor’s magnetic field, and *V_tr_*(*t*) is the harvester’s transducer voltage.

At the moment *t_o_*, when the switch opens, the energy stored in the inductor generates an induced voltage, *V_ind_*:

(6) $$V_{ind}=L_{r}{\frac{di_{L}\left(t\right)}{dt}|}_{t=t_{o}}$$

We can approximate the time derivation of the inductor current at the moment *t_o_* as:

(7) $${\frac{{di}_{L}\left(t\right)}{dt}|}_{t=t_{o}}=\frac{i_{L}\left(t_{O}\right)}{\Delta t}=\frac{\frac{1}{L_{r}}\int_{t_{C}}^{t_{O}}V_{tr}\left(t\right)dt}{\Delta t}$$

where *i_L_(t_o_)* is the inductor current at the instant of the switch opening, *t_c_* the time instant when the switch is closed, and Δ*t* is the time required for the inductor current to fall to zero.

If the voltages induced on the inductor are high enough to pass over the diodes, they will charge the output capacitor to the steady state voltage:

(8) $$V_{ro\_ss}=2\cdot \left(\frac{\int_{t_{C}}^{t_{O}}V_{tr}\left(t\right)dt}{\Delta t}-V_{D}\right)$$

where *V_D_* is the diode threshold voltage.

Neglecting energy losses, the maximal obtainable rectifier output voltage *V_ro_max_* depends on the inductance *L_r_*, capacitance *C_r_*_1,2_, and the current through the inductor at the instant the switch opens, *i_L_(t_o_)* (9) [21].

(9) $$V_{ro\_max}=i_{L}\left(t_{O}\right)\cdot \sqrt{\frac{L_{r}}{C_{r1,2}}}$$

The output capacitor *C_r_*_2_ gradually discharges when no signal is coming from the harvester’s transducer (as seen in Figure 2b) because of the leakage currents of the reversely polarized diodes, or the input impedance of the next interface stage.

## 3. Proposed Sensor Interface Characteristics and Design Parameters

**Figure 3:**
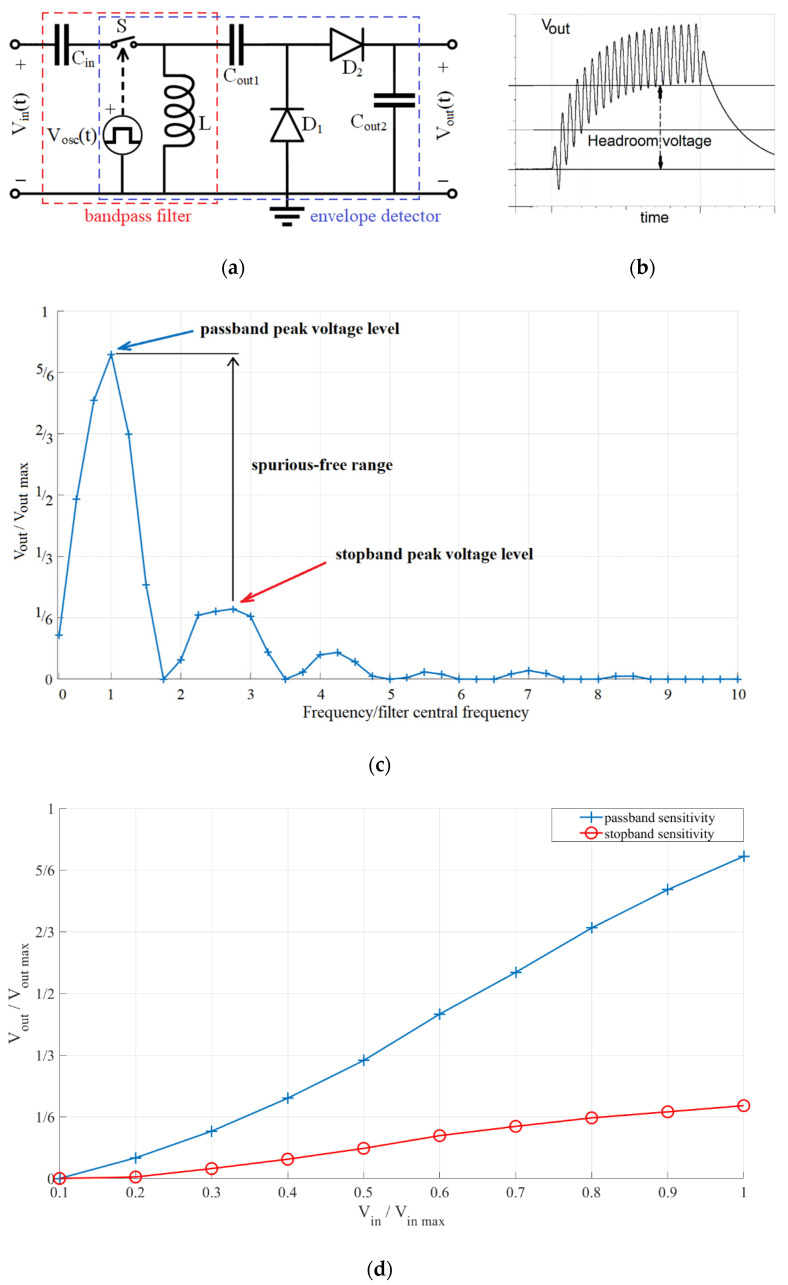
(**a**) Proposed sensor interface (with marked bandpass filter and envelope detector functional blocks), (**b**) output signal waveform (with marked headroom voltage), (**c**) normalized frequency characteristic and (**d**) normalized output headroom voltage with input voltage.

Combining the two functionalities explored in the literature, in this work we devise a low-power, frequency selective, voltage boosting sensor interface (Figure 3a), capable of operating with signals under 5 mV peak-to-peak and in the low acoustic frequency range, from 100 Hz to 1 kHz. For the interface to meet these demands, several of its characteristics should be considered. The first is the interface’s sensitivity (Figure 3c), the ratio of output headroom voltage and input voltage, with the headroom voltage defined as the voltage difference between the interface output voltage with no input and the interface lowest steady-state output voltage with a given input, as shown in Figure 3b. The stopband sensitivity should also be considered, as the maximal expected stopband voltage defines the lowest passband voltage levels with which the interface can operate (spurious-free range, Figure 3c). This leads to the next characteristic, the frequency selectivity, i.e., the difference between its passband and stopband sensitivities (Figure 3c,d). The final characteristic is the power consumption.

From the presented principles of operation and the desired characteristics of the proposed interface, we determined its key design parameters that can be divided in two groups: switch control signal parameters and passive component values.

The switch control signal parameters of interest are: switch control signal frequency, duty cycle, and delay between the switch control and input signal (the switch is controlled by an independent voltage signal *V_osc_(t),* as shown in Figure 3a).

The passive components of interest are: input capacitor *C_in_*, inductor *L*, *Q* factor of the input switched inductor filter, and output capacitors, *C_out_*_1_ and *C_out_*_2_, which we analyzed in detail in our previous work [17,18]. The diodes were also chosen based on previous work analyzing their influence on weak-signal rectifier performance [14,22].

## 4. Proposed Sensor Interface Simulation Study

### 4.1. Simulation Model

In order to both characterize the proposed sensor interface and narrow the parameter selection for the prototype realization, a SPICE model has been implemented and simulated in Texas Instruments’ PSpice (Dallas, Texas, TX, USA) following the schematic shown in Figure 3a. The obtained simulation results were further processed and presented using MathWorks’ MATLAB^®^ (Natick, Massachusetts, MA, USA).

The following parameters were varied to determine their influence on the output voltage characteristics and power consumption: switch control signal frequency and duty cycle, delay between the switch control signal and input signal, input capacitor, *C_in_*, inductor, *L*, and resistance, *R_L_,* filter quality factor, *Q*, defined as:

(10) $$Q=\frac{1}{R_{L}}\cdot \sqrt{\frac{L}{C_{in}}}$$

The output capacitors, *C_ou_*_1_ and *C_out_*_2_ were both 1 µF, following previous research conclusions and the diodes chosen for the simulation model were the HSMS-282x (Agilent Technologies, Santa Clara, California, CA, USA), because of their low forward voltage, low reverse current, and high saturation current. For simulation analyses showing the frequency characteristics, the input, *V_in_(t)*, was a sinusoidal signal with frequency varied from 50 Hz to 2000 Hz, with a 50 Hz step and 20 mV peak-to-peak, while the simulation analyses showing the sensitivity were done with an input sinusoidal signal of a fixed frequency in the range from 100 Hz to 600 Hz and voltage from 1 mV to 20 mV peak-to-peak with a 1 mV step.

### 4.2. Simulation Results

#### 4.2.1. Switch Control Signal Parameters—Duty Cycle and Frequency

**Figure 4:**
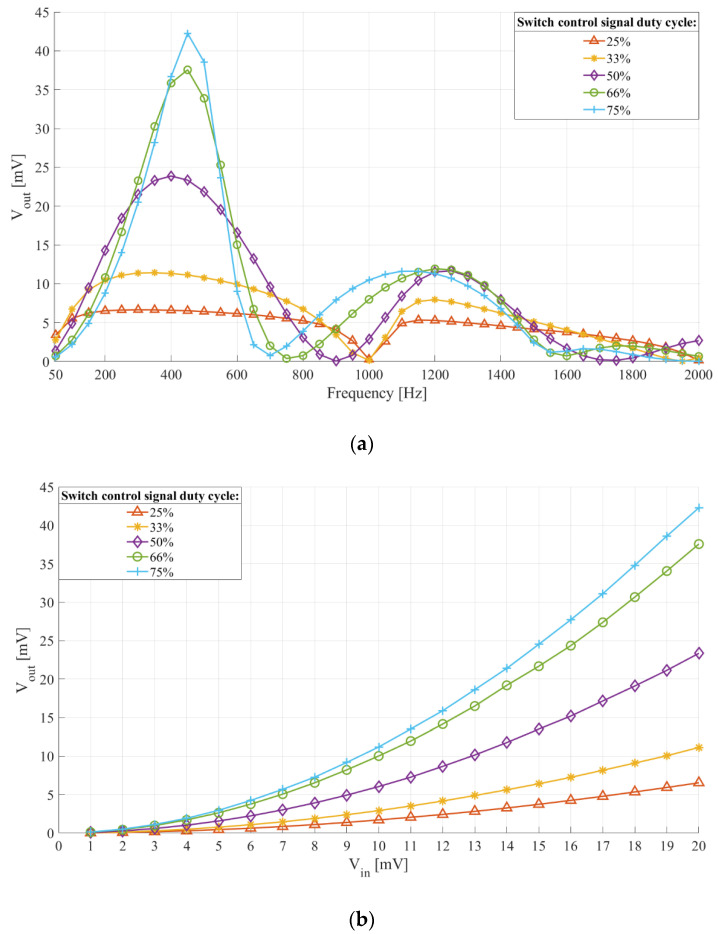
(**a**) Frequency characteristic and (**b**) output headroom voltage to input voltage relation of the sensor interface with switch control signal duty cycle. Filter central frequency 512 Hz (*L* = 100 mH, *C_in_* = 1 µF, *R_L_* = 66.6 Ω). Switch control signal frequency *f_switch_* = 1024 Hz and duty cycle from 25% to 75%. (**a**) Input voltage 20 mV peak-to-peak and frequency from 50 Hz to 2000 Hz with a 50 Hz step. (**b**) Input signal voltage from 1 mV to 20 mV with a 1 mV step. Input signal frequency 450 Hz.

Figure 4a,b show the interface frequency characteristic and the relation of output headroom voltage and input voltage with switch control signal duty cycle. The filter central frequency was 512 Hz (*C_in_* = 1 µF, *L* = 100 mH, *R_L_* = 66.6 Ω (Q = 4.8335)). The switch control signal frequency was 1024 Hz and duty cycles were 25%, 33%, 50%, 66%, and 75%.

From Figure 4a,b we see that increasing the switch control signal duty cycle leads to an increased sensitivity and a narrower frequency characteristic, both of which are desired traits. It also increases the central frequency of the interface passband towards the one of a fixed passive LC filter. These results adhere to the theoretical switched inductor filter performance presented in Section 2.1 and Figure 1b. We can also conclude that duty cycles under 50% should not be utilized, as they lead to low sensitivity and poorer frequency selectivity.

**Figure 5:**
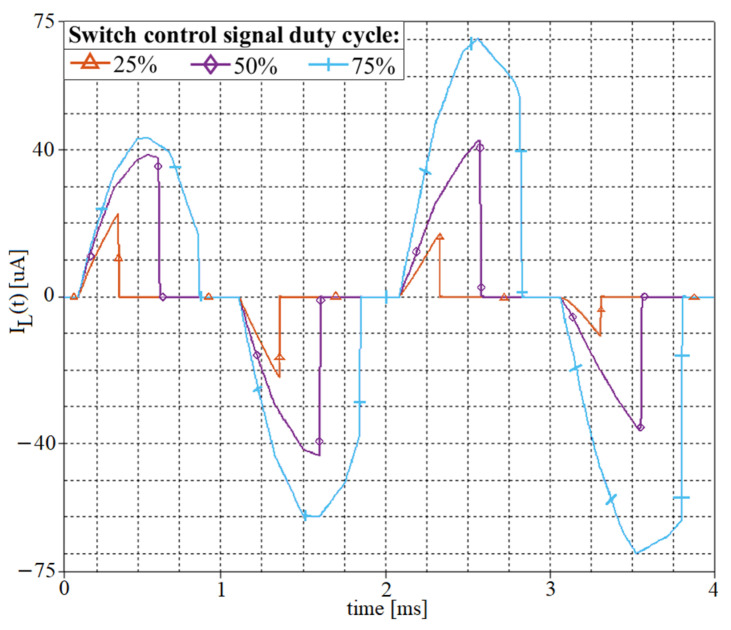
Inductor current of the sensor interface with switch control signal duty cycle. Filter central frequency 512 Hz (*L* = 100 mH, *C_in_* = 1 µF, *R_L_* = 66.6 Ω). Switch control signal frequency *f_switch_* = 1024 Hz and duty cycle 25%, 50%, and 75%. Input signal voltage 20 mV peak-to-peak and frequency 450 Hz.

However, increasing the duty cycle leads to longer periods of time in which the sensor drives the interface, leading to an increased sensor current. This is shown in Figure 5, which depicts the inductor current with switch control signal duty cycle. The simulation model was the same as for Figure 4a,b, and the switch control signal duty cycle was 25%, 50%, and 75%.

As we can see from Figure 5, both peak and mean inductor currents are determined by the switch control signal duty cycle. The peak and mean currents were around 23 µA peak and 2.88 µA mean for 25% duty cycle, 42 µA peak, and 10.5 µA mean for 50% duty cycle, and 70 µA peak and 26.25 µA mean for 75% duty cycle.

**Figure 6:**
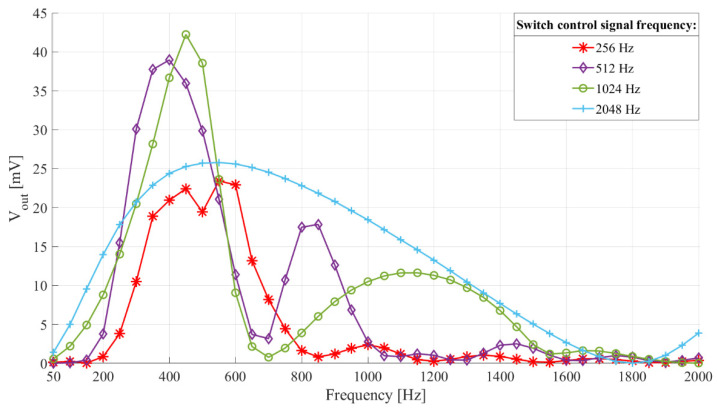
Frequency characteristics of the sensor interface with switch control frequency. Filter central frequency 512 Hz (*L* = 100 mH, *C_in_* = 1 µF, *R_L_* = 66.6 Ω). Input voltage 20 mV peak-to-peak and frequency from 50 Hz to 2000 Hz with a 50 Hz step. Switch control signal frequency, *f_switch_*, 256 Hz, 512 Hz, 1024 Hz, and 2048 Hz and duty cycle 75%.

Figure 6 shows the interface frequency characteristics with switch control signal frequency, *f_switch_*. The filter central frequency was 512 Hz (*C_in_* = 1 µF, *L* = 100 mH, *R_L_* = 66.6 Ω (*Q* = 4.8335)). The switch control frequency was 256 Hz, 512 Hz, 1024 Hz, and 2048 Hz. The switch control signal duty cycle was 75%.

Looking at Figure 6 we see that there is a switch control signal frequency that, with a given filter central frequency and *Q* factor, leads to the most frequency selective interface, with the highest sensitivity (in this case it is 1024 Hz, i.e., double the filter central frequency).

The dependency of the frequency characteristic and sensitivity on the switch control frequency can be explained by energy transfer from the input LC circuit to the output capacitors.

The maximal energy transfer occurs if the switch opens twice per inductor current period, precisely at maximal positive and negative inductor current values. The switch control signal frequency should be set slightly above double the frequency of the input signal of interest, to avoid the influence of time delay between the input and switch control signal on the output voltage (explained in the following text).

More than two switch openings per inductor current period cause more generations of induced voltage, but of lower value, which reduces the overall energy transfer efficiency, because of the exponential dependency of the diode current on the voltage on it, i.e., the induced voltage. Having more than two openings per input signal period also leads to a broader frequency characteristic (output voltage less dependent on the switch opening instant).

Finally, when considering the switch control signal parameters, it should be mentioned that the proposed sensor interface output voltage can also be influenced by the time delay between the input signal onset and the switch control signal. This effect explains the small discontinuities, like the one visible in Figure 6 at 500 Hz, on the red curve. However, this delay can substantially influence the interface output voltage only if the input signal frequency matches the switch control signal frequency or one of its specific rational multipliers (1/4, 1/2, 2, 3…). For all other input signals, this time delay can change the output voltage by no more than 10%. Therefore, this effect will not substantially impact the device’s application and performance with transducer inputs (which consist of frequencies of interest, other frequencies, noise, and interference).

#### 4.2.2. Passive Component Selection—Capacitor and Inductor

**Figure 7:**
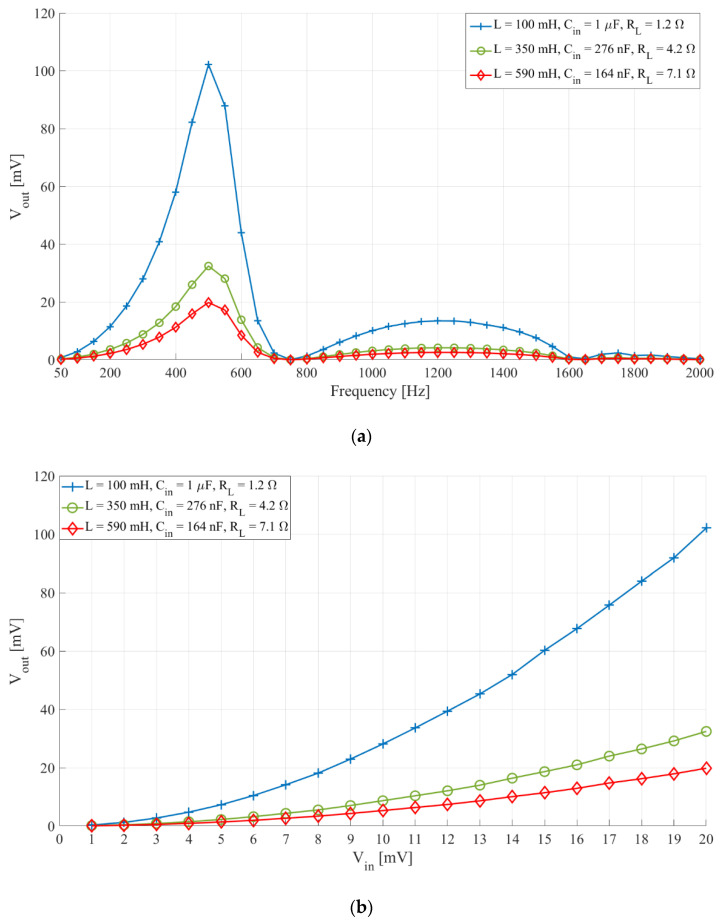
(**a**) Sensor interface frequency characteristic and (**b**) the relation of output headroom voltage and input voltage with *L*. The switch control frequency, *f_switch_*, 1080 Hz and duty cycle 75%, *Q* = 267.3. (**a**) Input signal voltage 20 mV peak-to-peak, frequency from 50 Hz to 2000 Hz with a 50 Hz step. (**b**) Input signal frequency 500 Hz and voltage from 1 mV to 20 mV peak-to-peak with a 1 mV step.

From Equation (1) it is clear that the same central frequency can be obtained with different values of inductance, *L*, and capacitance, *C*. This is shown in Figure 7a,b, which present the frequency characteristics and the relation of the output headroom voltage and input voltage of interfaces with different inductance and capacitance. The filter central frequency was 512 Hz, the switch control frequency was 1080 Hz, and the duty cycle was 75%. *L* were 100 mH, 350 mH, and 590 mH, and *C_in_*, were 1 µF, 276 nF, and 164 nF, respectively. The *Q* factor was kept constant (*Q* = 267.3) by setting the resistance, *R_L_*, to 1.2 Ω, 4.2 Ω, and 7.1 Ω, respectively.

From Figure 7a,b we see that interfaces with filters set to the same central frequency, have lower sensitivity the higher their inductance is. Furthermore, if we compare the results from Figure 4b with the results from Figure 7b we can see that an interface with a significantly lower *Q* factor (Figure 4b, *Q* = 4.8335) still has higher sensitivity than the two interfaces with higher inductances and higher *Q* factor (Figure 7b, *Q* = 267.3).

## 5. Proposed Sensor Interface Experimental Characterization

The goal of these measurements was to provide experimental verification of the simulation results and characterize the proposed sensor interface prototype in terms of frequency selectivity, sensitivity, and power consumption.

### 5.1. Measurement Setup

**Figure 8:**
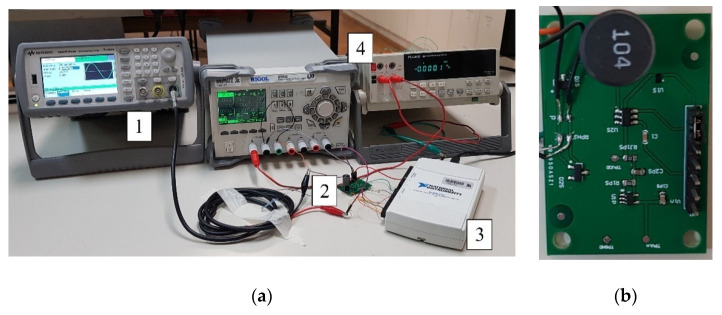
(**a**) A photograph of the measurement setup. (1) Keysight 33500B waveform generator, (2) sensor interface prototype, (3) NI USB-6211 data acquisition card. (4) Power supply (RIGOL DP832) and a multimeter for supply current measurement (Fluke 45). (**b**) Proposed frequency-selective voltage-boosting sensor interface prototype.

Figure 8a shows a photograph of the measurement setup. The measurement setup consisted of a Keysight 33500B waveform generator (Keysight Technologies, Santa Rosa, California, CA, USA) for generating the input and switch control signal, the prototype sensor interface (shown in Figure 8b), and an NI USB-6211 (National Instruments, Austin, Texas, TX, USA) data acquisition card connected to a PC for recording the output voltage. The power consumption of the interface was measured using a Fluke 45 multimeter (Fluke Corporation, Everett, Washington, WA, USA). The interface was powered by a DP832 power source from RIGOL (RIGOL Technologies, Beijing, China).

The prototype of the proposed frequency-selective voltage-boosting sensor interface was designed according to the schematic in Figure 3a, with components shown in Table 1.

### 5.2. Measurement Procedure and Results

The measurement results were recorded by a National Instruments NI USB-6211 data acquisition card. The data acquisition control, data processing, and presentation were implemented using MathWorks’ MATLAB^®^ (Natick, Massachusetts, MA, USA).

#### 5.2.1. Frequency Selectivity

The goal was to characterize the sensor interface frequency selectivity with different input capacitors, *C_in_*, inductors, *L*, and switch control signal frequencies.

The filter central frequencies were: 139 Hz (*C_in3_*, *L*_2_), 211 Hz (*C_in_*_2_, *L*_2_), 512 Hz (*C_in_*_2_, *L*_1_), and 655 Hz (*C_in_*_1_, *L*_2_). The switch control signal duty cycle was 50% and the frequency was 256 Hz, 278 Hz, 422 Hz, 512 Hz, 1024 Hz, and 1310 Hz. The input signal voltage was 20 mV peak-to-peak and the frequency was ranging from 50 Hz to 2000 Hz, with a 50 Hz step.

**Figure 9:**
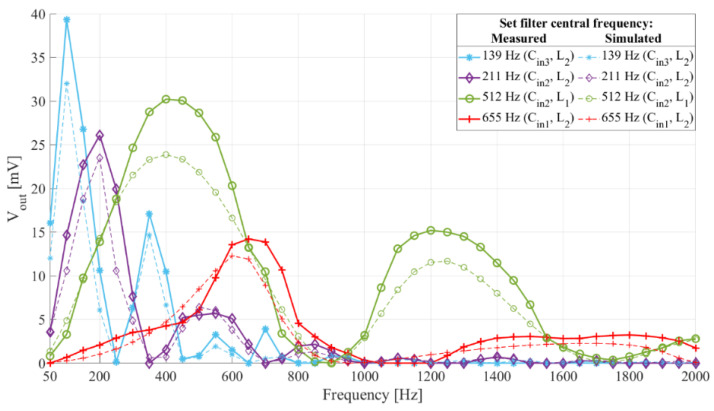
Frequency characteristic of the sensor interface prototype with different *C_in_*, *L* and *R_L_*. The switch control duty cycle 50%. The switch control frequencies were 278 Hz (blue), 422 Hz (purple), 1024 Hz (green), and 1310 Hz (red). The dashed lines show simulation results, paired with the experimental results by color and same markers.

**Figure 10:**
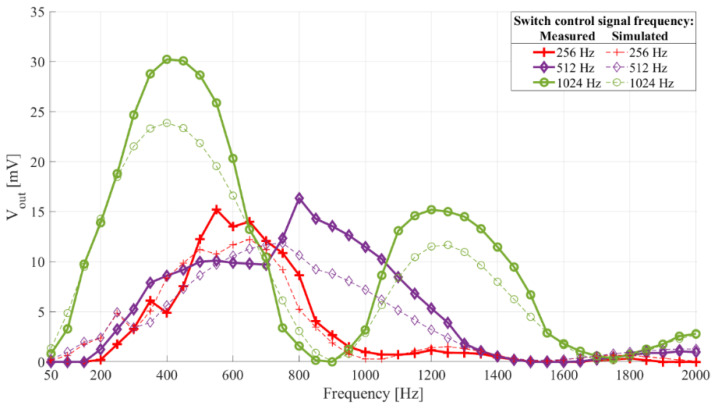
Frequency characteristic of the sensor interface prototype with the filter central frequency 512 Hz (*C_in_*_2_, *L*_1_). Switch control duty cycle 50%, frequency 256 Hz, 512 Hz, and 1024 Hz. The dashed lines show simulation results, paired with the experimental results by color and same markers.

Figure 9 shows the frequency characteristics of the interface with four filter central frequencies and Figure 10 shows the frequency characteristics of an interface with a filter central frequency of 512 Hz and three different switch control signal frequencies. In addition to the measurement results, both figures show the simulation results for the same setups.

Comparing the experimental and simulation results shown in Figure 9 and Figure 10, we see the frequency characteristics of the prototype interface match those of the simulated interfaces.

In [6,23] the authors presented the idea of reducing power consumption of wake-up interfaces and increasing their flexibility with reconfigurability, while in [10] digital setting of the filter central frequency was presented as an interesting feature for a wake-up interface. From the results in Figure 9, the filter central frequency of this interface can be digitally set by simultaneously selecting the input capacitor and switch control frequency. The settling time of the reconfigurable switched inductor circuit can be shorter than of that of a circuit utilizing an operational amplifier-based active bandpass filters.

#### 5.2.2. Sensitivity

The goal was to determine the sensor interface sensitivity with different filter central frequencies (different input capacitors, *C_in_*, and inductors, *L*) and switch control signal frequencies.

The prototype setup was identical to the one described for frequency selectivity measurement. The input signal frequency was 100 Hz, 200 Hz, 450 Hz, and 650 Hz and, the voltage was ranging from 2 mV peak-to-peak to 20 mV peak-to-peak with a 2 mV step.

**Figure 11:**
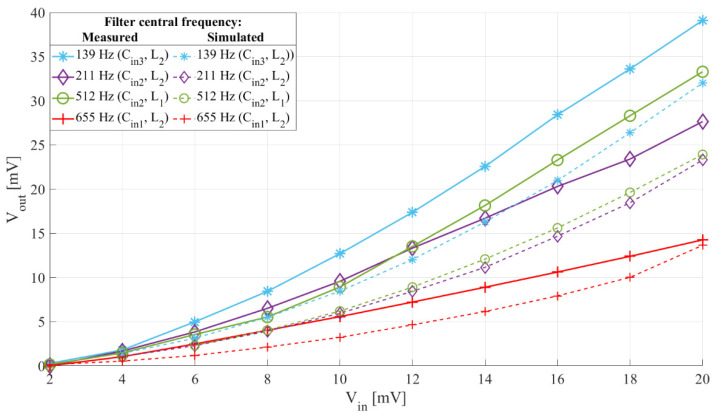
Sensor interface output headroom voltage to input voltage relation with different *C_in_*, and *L*. Switch control frequency 278 Hz (blue), 422 Hz (purple), 1024 Hz (green), and 1310 Hz (red), and duty cycle 50%. Input signal frequency 100 Hz (blue), 200 Hz (purple), 450 Hz (green) and 650 Hz (red), and voltage from 2 mV to 20 mV peak-to-peak with a 2 mV step. The dashed lines show simulation results, paired with the experimental results by color and same markers.

**Figure 12:**
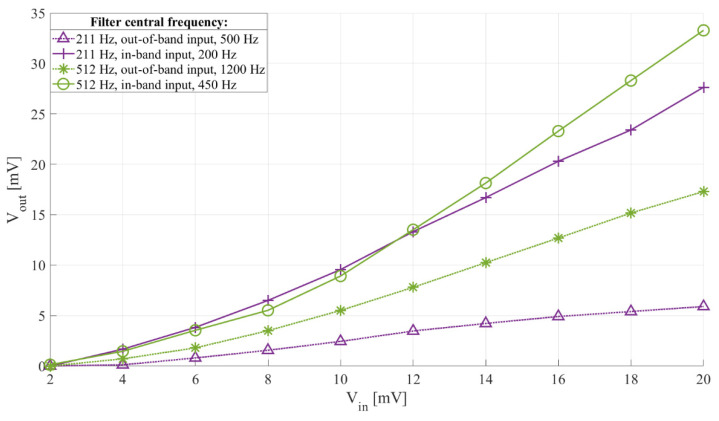
Sensor interface passband and stopband output and input voltage relation with *C_in_*, and *L*. Filter central frequency 211 Hz (*C_in_*_2_, *L*_2_) and 512 Hz (*C_in_*_2_, *L*_1_). The switch control frequency 422 Hz and 1024 Hz, respectively, and the duty cycle 50%. Input signal frequency 200 Hz and 500 Hz for the 211 Hz interface, and 450 Hz and 1200 Hz for the 512 Hz setting, and voltage from 2 mV to 20 mV peak-to-peak with a 2 mV step.

Figure 11 shows the measured and simulated output headroom voltage with input voltage of the interface whose frequency characteristics are shown in Figure 9, while Figure 12 shows the measured passband and stopband output-to-input voltage relation of two setups of the interface, with an input signal frequency of 200 Hz and 500 Hz for the 211 Hz filter central frequency setup, and 450 Hz and 1200 Hz for the 512 Hz filter central frequency setup.

From Figure 11 we can see that the sensitivity of the proposed sensor interface reaches up to approximately 2 mV/mV and it can be adjusted by choosing the input capacitor, *C_in_*, inductor, *L*, and switch control signal frequency. Comparing the experimental and simulation results shown in Figure 11, we see that the sensitivities and their trends of the developed prototype match those of the simulated interfaces.

From Figure 12 we can see that when the interface filter is set to 512 Hz, the interface has a higher passband sensitivity (around 1.66 mV/mV) than when it is set to 211 Hz (around 1.38 mV/mV), but also, due to the lower *Q* factor, it leads to a higher stopband sensitivity (0.87 mV/mV compared to around 0.3 mV/mV), making the 211 Hz interface setting more than twice more frequency selective than the 512 Hz one.

#### 5.2.3. Power Consumption

The goal was to determine the power consumption of the proposed sensor interface prototype with selected components: the 1024 Hz SiT1569 oscillator, the input capacitor *C_in_*_2_ and inductor *L*_1_. The power consumption was determined by multiplying the interface supply voltage of 1.8 V with its supply current, measured by a Fluke 45 multimeter.

The measured interface current consumption, consisting of the oscillator and switch current consumptions, was 1.84 µA, with a 1.8 V power supply, resulting in a power consumption of 3.31 µW.

The overall power consumption was predominantly defined by the oscillator, further emphasizing the crucial role of the switch control signal generator selection in achieving low power consumption.

## 6. Design Recommendations

Following the numerical and experimental analyses of the interface’s functionality and design parameters, this section presents a set of recommendations for interface synthesis.

The interface synthesis is performed in a series of steps:

1. Choosing the output capacitors *C_out_*_1_ and *C_out_*_2_ to ensure the desired output signal waveform and its key parameters (more details in [17,18]).
2. Determining the wanted frequency characteristic of the interface, by choosing its resonant frequency, *f_res_*, (and angular frequency *ω_res_* = 2π *f_res_*) and *Q* factor. This choice is made considering the frequency characteristic of the input signal of interest.
3. Setting the desired sensitivity at the resonant frequency.

When considering Equation (9) for determining the maximal obtainable output voltage of the proposed interface, the inductor current can be expressed using the input voltage and input circuit impedance, *Z_in_*:

(11) $$V_{out}=\frac{V_{in}}{\left|Z_{in}\right|}\sqrt{\frac{L_{r}}{C_{out1,2}}}$$

with *Z_in_* given as:

(12) $$\left|Z_{in}\right|=\sqrt{{\left(\omega_{in}\cdot L-\frac{1}{\omega_{in}\cdot C_{in}}\right)}^{2}-R_{L}{}^{2}}$$

where *ω_in_* is the input signal angular frequency.

From this, we can get an expression for the maximal sensitivity (at the resonant frequency):

(13) $$\frac{V_{out}}{V_{in}}=\frac{1}{\sqrt{L}}\cdot \frac{Q}{\omega_{res}\cdot \sqrt{C_{out1,2}}}$$

where *ω_res_* is the input circuit resonant angular frequency, and *Q* the quality factor, given in Equation (10).

From Equation (13), it is clear that, with a chosen output capacitance, input circuit resonant frequency and *Q* factor, the interface sensitivity at the resonant frequency is set by choosing the appropriate inductance value.

4. Setting the switch control signal duty cycle to 50%, as this provides a suitable sensitivity, frequency selectivity and power consumption. Small increases of the duty cycle can be considered for slight central frequency tuning, despite of increasing the design complexity, but not over 60%, due to increased power consumption.
5. Setting the switch control signal frequency, *f_switch_*, to around 2% to 5% higher than double of the frequency of the input signal of interest.

(14) $$f_{switch}=\left(1.02\;\sim \;1.05\right)\cdot 2f_{in}$$

To conclude this set of design guidelines, an exemplary evaluation of the maximal obtainable sensitivity is shown for one interface setup utilized in the experiments and simulations. With a filter central frequency of *f_res_* = 512 Hz, a *Q* factor of around 4.8, an inductor of *L* = 100 mH, and output capacitors of *C_out_*_1,2_ = 1 µF, we get a maximal obtainable interface sensitivity of around 4.7 mV/mV.

## 7. Functional Test and Comparison

### 7.1. Measurement Setup

The measurement setup for comparison of the proposed interface and one consisting of an active bandpass filter and a passive voltage doubler [9] was the same as for the experimental characterization of the proposed sensor interface (Figure 8a).

The proposed sensor interface’s (Figure 3a) filter central frequency was 512 Hz with a 400 Hz bandwidth (*C_in_* = 1 µF, *L* = 100 mH, *R_L_* = 66.6 Ω). Its switch control signal duty cycle was 50% and the frequency was 1024 Hz. The output capacitors, *C_out_*_1_ and *C_out_*_2_*,* were 1 µF, and the HSMS-282x diodes, the TMUX1101 switch, and the SiT1569 1024 Hz oscillator were used.

**Figure 13:**
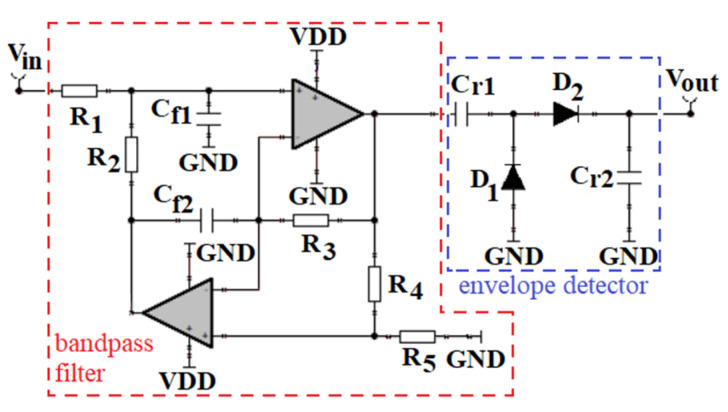
Schematic of the sensor interface consisting of an active GIC bandpass filter and a passive two-diode voltage doubler [9,18].

It was compared to a sensor interface consisting of an active general impedance converter (GIC) bandpass filter and a passive two-diode voltage doubler, as shown in Figure 13 [9,18]. The filter central frequency was 500 Hz, with a passband bandwidth of around 300 Hz. The rectifier capacitors *C_r_*_1_ and *C_r_*_2_ were 22 nF, to allow the capacitor to fully charge and achieve maximal headroom voltage during each event of interest [17,18].

### 7.2. Measurement Procedure

The two compared interfaces consist of the same two functional blocks (bandpass filter and envelope detector) and perform the same function of frequency signal decomposition and envelope extraction. To establish if the previously developed interface can be replaced by the one proposed in this work, a comparison of their output headroom voltages (Figure 3b) was performed, using a prerecorded speedboat signal input (twin-engine speedboat passing over a hydrophone submerged approximately 1 m under the surface in shallow water) [24].

**Figure 14:**
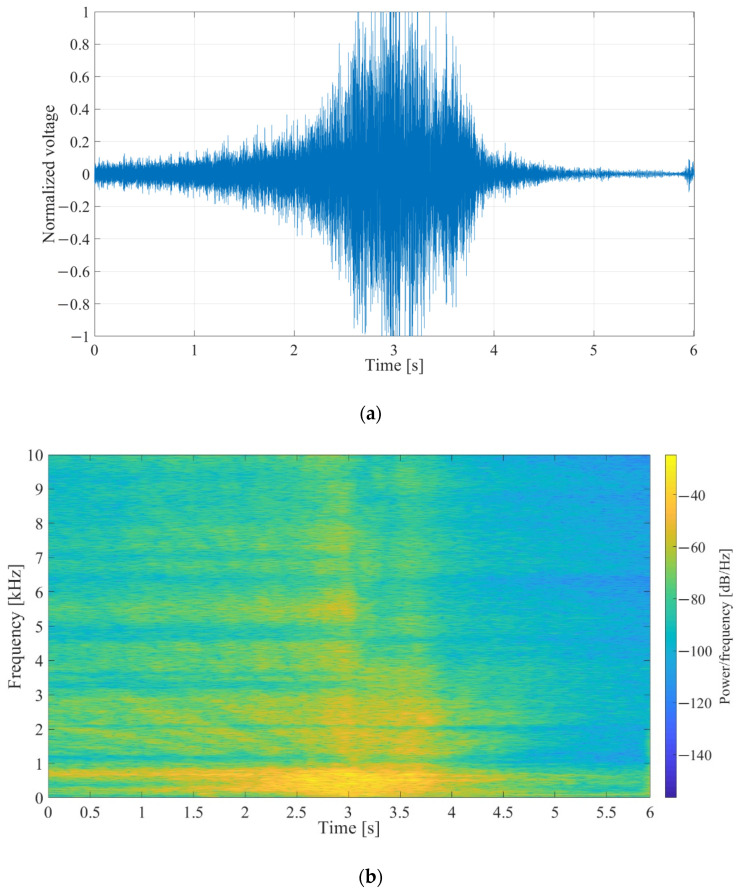
(**a**) Waveform of the prerecorded input signal, *V_in_*, with a duration of approximately 3 s, followed by around 3 s of pause. The voltage shown was normalized with regards to maximal value. (**b**) Spectrogram of the prerecorded input signal [18].

The signal waveform with normalized amplitudes and its spectrogram are shown in Figure 14a,b, respectively. The input signal is periodical, each period consisting of approximately 3 seconds of the passing speedboat, followed by around 3seconds of pause. The maximal input signal voltage was scaled from 2 mV to 20 mV peak-to-peak, in steps of 2 mV.

### 7.3. Results

**Figure 15:**
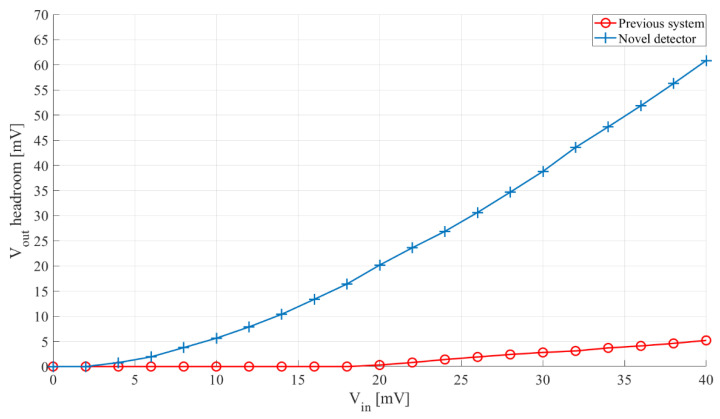
Comparison of outputs of the proposed sensor interface and one consisting of a bandpass filter and a passive voltage doubler. Input—prerecorded speedboat signal, 3 s of signal, 3 s of pause, scaled from 0 mV peak-to-peak to 40 mV peak-to-peak.

The two sensor interfaces’ comparison with the prerecorded speedboat signal input is shown in Figure 15.

The results show that the proposed sensor interface outperforms the previously developed one, being able to operate with signals around 5 mV peak-to-peak, while the previously developed one required over 20 mV peak-to-peak. The 1.5 mV/mV sensitivity of the proposed interface stems from the increased rectification efficiency provided by the switched inductor.

In addition to the mentioned improvements, it should also be noted that the proposed sensor interface has a power consumption of 3.31 µW compared to 8.25 µW consumed by the previously developed one, which represents a reduction of around 60%. This means that replacing the previously developed interface with the interface proposed in this work, would either extend the sensor node life-time, or allow for more sensors with the same power budget, leading to increased event detection accuracy.

To conclude the demonstration of applicability of the proposed interface in low-power analog acoustic event detection, Table 2 shows a comparison of its functionality and power consumption to state-of-the-art similar interfaces.

## 8. Conclusions

Low-power analog sensors and interfaces present a necessity in IoT development. Following previous research on switched inductor filters and energy harvesters, a novel switched inductor frequency selective sensor interface is proposed. A simulation study was done to determine the key design parameters and characterize the interface performance with input signals up to 20 mV peak-to-peak, at low acoustic frequencies from 100 Hz to 1 kHz. A prototype interface was developed and characterized, achieving the maximal sensitivity of approximately 2 mV/mV in the passband, four times lower sensitivity in the stopband, and a power consumption of approximately 3.31 µW. The novel sensor interface can operate with inputs around 5 mV compared to over 20 mV needed for the one consisting of an active bandpass filter and a passive voltage doubler, having around 60% lower power consumption (3.31 µW compared to 8.25 µW), thus enabling life-time extension or improved detection. The future work will focus on reconfigurable switched inductor sensor interfaces and lowering the power consumption of the switch control oscillator.

## Figures and Tables

**Table 1:** Prototype components.

*Integrated Components*				
Component	Manufacturer	Supply Voltage	Supply Current (Typical)	Transition Times (Typical)
switch TMUX1101	Texas Instruments	1.8 V	3 nA	12 ns
oscillator SiT1569	SiTime	1.8 V	1.7 µA–3.3 µA	200 ns
***Discrete Semiconductor Components***				
**Component**	**Manufacturer**	**Reverse Current (at 1V)**	**Saturation Current**	**Forward Voltage (Maximal)**
diodes HSMS-282x	Agilent	100 nA	22 nA	0.34 V
***Discreet Passive Components***				
**Component**		**Value**		**Type**
Output capacitors		*C_out_1__* = *C_out_*_2_ = 1 µF		Multilayer ceramic
Input capacitors		*C_in_1__* = 100 nF, *C_in_*_2_ = 1 µF, *C_in3_* = 2.2 µF		Multilayer ceramic
Inductors		*L*_1_ = 100 mH, *R_L_*_1_ = 66.6 Ω		Air-core
		*L*_2_ = 590 mH, *R_L_*_2_ = 7.1 Ω		Ferrite-core

**Table 2:** Per channel power consumption comparison of state-of-the-art acoustic event detector sensor interfaces with the proposed interface.

Reference	Technology	Functionality	Power Consumption (µW)
This work	Embedded design, COTSC	frequency decomposition and envelope detection	3.31
[10]	Embedded design, COTSC	frequency decomposition, envelope detection, 1-bit quantization (adjustable)	22.59
[9]	Embedded design, COTSC	frequency decomposition, envelope detection, 1-bit quantization (adjustable)	11.52
[25]	Embedded design, COTSC	frequency decomposition, amplification, template matching (adjustable)	9.32
[6]	Custom FPAA	frequency decomposition, amplification, peak detection, quantization, pattern recognition (programmable)	5.38
[26]	ASIC	energy threshold detection, 16 feature extraction based on amplification, filtering and absolute value detection, and classification	6
[27]	ASIC	frequency decomposition, magnitude detection, quantization, template matching	2.92

COTSC—commercial of-the-shelf components; FPAA—field-programmable analog array; ASIC—application specific integrated circuit.

## Data Availability

Not applicable.
